# Best Practices for Treating Blind and Visually Impaired Patients in the Emergency Department: A Scoping Review

**DOI:** 10.5811/westjem.61686

**Published:** 2024-05-03

**Authors:** Kareem Hamadah, Mary Velagapudi, Juliana J. Navarro, Andrew Pirotte, Chris Obersteadt

**Affiliations:** * University of Kansas School of Medicine, Kansas City, Kansas; † University of Kansas Health System, Kansas City, Kansas; ‡ University of Kansas Medical Center, Kansas City, Kansas; § University of Kansas Medical Center, Department of Emergency Medicine, Kansas City, Kansas; ¶ University of Kansas Medical Center, Office of Student Affairs, Kansas City, Kansas; # Rockhurst University, Kansas City, Kansas

## Abstract

**Introduction:**

Blind and visually impaired individuals, an under-represented population of the emergency department (ED), possess comorbidities and have a higher chance of in-hospital sequelae, including falls. This potentially vulnerable population, if not treated mindfully, can be subject to decreased quality of care, recurrent and/or longer hospitalizations, persistence of health issues, increased incidence of falls, and higher healthcare costs. For these reasons, it is crucial to implement holistic practices and train clinicians to treat blind and visually impaired patients in the ED setting.

**Methods:**

We identified and used a comprehensive article describing best practices for the care of blind and visually impaired patients to establish the ED-specific recommendations presented in this paper. A scoping review of the literature was then performed using PubMed to identify additional articles to support each recommendation. To ensure that recommendations could be implemented in a representative, scalable, and sustainable manner, we consulted an advocate for the blind to help refine and provide additional suggestions.

**Results:**

We identified 14 recommendations that focus on communication strategies, ED resource access, and continuity of care. The main recommendation is for the clinician to support the unique healthcare needs of the visually impaired individual and maintain the patient’s autonomy. Another recommendation is the consistent use of assistive devices (eg, canes, guide dogs) to aid patients to safely ambulate in the ED. Also identified as best practices were discharge education with the use of a screen reader and timely follow-up with a primary care physician.

**Conclusion:**

While we summarize a variety of recommendations in this article, it is important to implement only the strategies that work best for the patients, personnel, and environment specific to your ED. After implementation, it is vital to refine (as frequently as needed) the interventions to optimize the strategies. This will enable the provision of exceptional and equal care to blind and visually impaired patients in the ED.

## INTRODUCTION

The blind and visually impaired (VI) are a small but highly marginalized population in the United States and around the world.^1^^,^^2^ There are approximately nine million VI people in the US, with blind people making up slightly less than 1% of the population. Globally, about 2% of children are considered VI.^3^ Estimates in the US are expected to double by 2050, with the VI population projected to be more prevalent in racial minorities and in the southern US. The reason for this increase is multifactorial but may be due to an ever-increasing aging population and differential access to preventative services among minority groups.^4^^,^^5^ Data is limited on the exact number of VI patients who are seen in the emergency department (ED). However, approximately 0.2% of patients admitted to US hospitals are considered VI.^6^ States that have not expanded Medicaid coverage see higher rates of VI patients in their EDs.^7^ Patients with other disabilities, such as those in the deaf and hard-of-hearing (DHH) community, are also more likely to seek ED care compared to those not in the DHH community.^8^^–^^12^

Although VI patients represent a relatively small proportion of patients seen in the ED and admitted to the hospital, they have significantly worse outcomes: They are admitted and readmitted more often, incur higher healthcare costs, and may have a higher in-hospital mortality rate.^6^^,^^13^^,^^14^ Visually impaired patients are more likely to experience multiple morbidities, thus further increasing their risk of needing ED care.^15^ Falls and their sequelae, such as hip fractures, are among the most common reasons for blind patients to be seen in the ED.^16^^–^^18^ Pediatric VI patients are particularly likely to incur orthopedic injuries and are also more likely to have fractures upon presentation.^19^ Hospitalized patients who are VI are also more likely to experience delirium,^20^ a well-known risk factor for morbidity and mortality.^21^ These injuries and conditions among the VI occur in other countries^22^ and to other disabled groups.^12^^,^^23^ In the US, these issues are further compounded by the intersections of race and age.^2^ Black patients and patients insured by Medicare (ie, those ≥65 years old) are the most likely to have extended hospital stays.^13^

Optimal care for all patients in the ED remains an ongoing challenge; care of VI patients presents unique challenges that offer a number of opportunities. A mindful approach to care of VI patients requires that EDs and clinicians pursue best practices, support staff, impactful education, and specialized considerations. As with many populations, the needs of VI patients impact their experience during ED care. In this article, we present best practice considerations. This scoping review is intended to prompt improved practice and to further discussion to optimize ED care for VI patients.

## METHODS

We performed a scoping review to identify PubMed articles related to blindness and VI in the ED, with particular emphasis on assessing the experiences of VI patients in the ED. Articles were included if they met one or more of the following criteria: centered on the experiences of disabled people, particularly VI people, in healthcare; discussed the experiences or epidemiology of disabled patients in EDs or hospitals regardless of geographic region; provided best practice recommendations for the care of VI patients regardless of specialty; and discussed outcomes of disabled patients in the ED or hospital. We excluded articles discussing the care of acute blindness or VI, as the focus of this review was on patients with pre-existing visual impairment. Due to the overall lack of data on this topic, guidelines from other specialties (eg, ophthalmology) were included and adapted to the ED setting.

We used the Americans with Disabilities Act (ADA) Checklist by Marshall and Joffee (2006) as the basis of our recommendations, as it provides a comprehensive list of best practices for all healthcare clinicians. From this paper, we selected 15 recommendations most relevant and applicable to the ED setting (Table). Recommendations were supplemented using focus group and survey data found on PubMed. The search phrase “(visually impaired) AND (accessibility)) AND (emergency department)” resulted in 28 results. We found one relevant study by Carmichael et al (2023), in which 12 disabled individuals were interviewed (six of whom were VI). Due to a lack of data specific to VI patients, the search was expanded to include the experiences of patients with other physical and cognitive disabilities, which yielded an additional study by Morris et al (2021).

**Table.** Summary of recommendations for interacting with visually impaired patients in the emergency department.

Recommendations	Rationale	References
Use optimal language: disability-first often preferred.	Better represents the patient’s lived experiences	24–27, 32
Introduce yourself every time you enter the room (consider placing signage to alert staff).	Ensures patient is aware of who is in the room at all times and may help prevent delirium	28–30, 32
Tell the patient what you’re going to do before doing it, including before leaving the room.	Ensures maximal patient autonomy and may help prevent delirium	29, 31–35
Listen to the patient’s caregiver(s), if applicable, but only after gathering as much information from the patient as you can.	Caregivers can provide important insight into the patient’s life	32, 36
If available at your facility, ask whether the patient would like an advocate.	VI patients are part of a socially and medically vulnerable community	32, 33, 37, 38
Accommodate the needs of the patient, but do not over-focus on visual impairment during the HPI.	Most VI patients do not present for concerns associated with their VI	29, 31–33, 36, 39, 40
Place the patient in quietest part of the ED.	May help prevent delirium	32, 35, 41–43
Ensure the patient has access to mobility equipment (eg, cane, guide dog) at all times.	Ensures maximal patient autonomy and may help prevent delirium	29, 32, 33, 35
Ensure the patient has access to personal technology (eg, phone, smartwatch, etc).	Ensures maximal patient autonomy and may help prevent delirium	30, 32
Ensure the patient knows where the call light is and how to use it.	Ensures maximal patient autonomy and may help prevent delirium	30, 32
Use the correct strategies when guiding a patient.	Helps ensure patient safety	31, 32
Clearly note the patient’s visual impairment in the medical record (ICD-9: 369; ICD-10: H54).	Helps ensure all healthcare workers are aware of the patient’s VI and can provide relevant accommodations	30, 32
Advocate for Medicaid expansion at the state and medical society (eg, AAEM) level, and encourage patients to apply.	May help decrease frequency of ED visits	7, 32
Help the patient establish care with a PCP.	Helps to prevent recurrent ED visits	32, 44, 45

*HPI*, history of present illness; *ED*, emergency department; *VI*, visually impaired; *ICD*, International Classification of Diseases, Rev 9 or 10; *AAEM, American Academy of Emergency Medicine; PCP, primary care physician.*

We evaluated trends in ED use among disabled patients to contextualize the recommendations provided. Finally, we used articles by the National Federation of the Blind (NFB) to ensure that the voices of VI authors and academics were well represented and to inform several recommendations (eg, language). Most of the data were observational and retrospective. We also consulted a subject matter expert who was born blind and dedicated her career to advocating for other VI people to ensure that we were best representing the needs of VI patients. Using this data, we identified actionable recommendations and best practices.

## RESULTS

We performed PubMed searches to identify supporting articles for all 14 recommendations (see Table). Articles were selected using the previously described inclusion and exclusion criteria. Excluding the ADA Checklist by Marshall and Joffee, which was used to develop each recommendation, we found four articles supporting recommendation one. Three articles were found supporting recommendation two. Five articles were found supporting recommendation three, and one article was found supporting recommendation four. We found three articles supporting recommendation five, six articles supporting recommendation six, and four articles supporting recommendation seven. Three articles were found supporting recommendation eight. One article was found supporting recommendation 9–13. Finally, we found two articles supporting recommendation 14. All supporting articles and which recommendations they informed can be found in Table.

## DISCUSSION

### Communication Strategies

#### Optimal Language

The use of person-first (eg, person who is blind) and disability-first (eg, blind person) language is a contentious issue. Academics consider person-first language to be more dignifying as it places focus less on the disability and more on the individual.^24^^,^^25^ However, many blind people and blind advocates strongly disagree with person-first language as it may inadvertently stigmatize disability. Blind advocates also argue that disability-first language more accurately represents disabled experiences.^25^^–^^27^ This contention further emphasizes the importance of listening to disabled patients and using the terminology they prefer. If a blind patient prefers to be called a “blind patient” or a “patient who is blind,” that preference should be accommodated like any other. Disability-first language will be used in this paper for brevity and, more importantly, because it is generally preferred by the VI community.

#### Entering and Exiting

Consent is an integral component of patient care, and all efforts should be made by emergency clinicians and patient care staff to obtain informed consent at all times.^28^ However, the way that consent is obtained cannot be uniformly applied to all patients. For example, blind patients cannot see who is entering their room, so they may not immediately be able to tell whether the person who just walked in is a doctor, nurse, family member, etc. Thus, it is imperative for each person entering a blind patient’s room to verbally inform the patient of their name and role every time they enter the room.^29^ This is especially important in the ED, an often hectic and disorienting place for all patients, and particularly for those with disabilities.^30^ Just as important as announcing when you walk into a patient’s room is announcing when you or others involved in patient care leave the room.^29^ If this is not done, the patient may attempt to speak to someone who they logically assume is still in the room only to be met with silence. This is not only potentially embarrassing but disorienting.^31^^,^^32^

#### Informed Consent

Informed consent discussions also must be tailored for VI patients. In addition to the typical discussions to gain consent, VI patients benefit from the clinician maintaining an ongoing dialogue during a procedure, explaining what will be done next and providing clear, actionable instructions when necessary.^29^^,^^33^ Adding this extra layer of communication can be instrumental in ensuring patient safety and adherence, and the overall efficacy of the medical intervention for blind patients. Furthermore, it serves to maintain respect for their autonomy, helps foster a cooperative environment, and minimizes surprise or discomfort during the procedure, a particularly important consideration in an ED setting where the pace of care is often rapid and potentially anxiety-inducing.^34^^,^^35^

### Mindfulness of Unique Needs

#### Navigating Caregivers

If a caregiver is not present, you may ask the patient or check the patient’s chart for a potential caregiver’s contact information. However, do not assume a patient has or requires a caregiver because they are VI. During the course of treatment of a VI patient, the caregiver (if applicable) may be able to provide helpful information or context regarding the patient.^36^ For example, the caregiver may provide information about the patient’s baseline independence and Activities of Daily Living—the skills needed to independently care for oneself. This information can be helpful during the course of treatment in the ED, as well as upon discharge to customize instructions to the patient. However, it is important to remember that caregivers are an adjunct to patient care and not the patients themselves. Thus, be sure to gather as much information from the patient as possible as well as from their caregiver.^29^^,^^32^ This helps maintain a respectful and autonomous patient-clinician relationship.

#### Using a Patient Advocate

Patient advocates can play a significant role in the holistic care of a patient.^37^^,^^38^ During the course of treatment for a VI patient, it is important to ask the patient whether they have an advocate, which can be done as early as the triage process. If the patient does not already have an advocate or cannot think of someone, it is important to work collaboratively with the patient to identify an advocate, if they would like one. There are several potential people who can be advocates including family and friends of the patient, work colleagues, caregivers, social workers, and hospital volunteers (eg, premedical students and navigators).^32^^,^^33^

The role of an advocate may vary; therefore, it is critical to establish clear roles and responsibilities for the advocate. One of their key responsibilities can be to accompany the patient in the waiting room. If the advocate is an employee of the hospital or familiar with the ED, it can be helpful for the advocate to discuss the overall ED process. This will provide predictability of what to expect and clarify the ED process for the patient.^37^ After the waiting room, the advocate can also provide support during transport to the room and in meeting healthcare personnel and explaining the work up and procedures for labs or imaging. Finally, during disposition, the advocate can appropriately advocate on behalf of the patient for resources required following discharge or during the admission process. The overall roles and responsibilities can vary by patient and ED setting, but it is important for the patient and the advocate to establish a mutual understanding.

#### History of Present Illness Considerations

When gathering the history of present illness (HPI) on a VI patient, emergency clinicians should strive to treat the patient as similarly to other patients as possible. For example, looking at the patient directly when you are speaking, as you would for other patients, is considerate and thoughtful.^29^^,^^31^^–^^33^ It is also important to recognize that VI exists on a spectrum from slightly decreased visual acuity to a complete lack of vision, and most people typically considered blind have some level of visual function.^39^ Acknowledging this spectrum, clinicians should attempt to discern the patient’s unique needs to provide optimal care. It is also important *not* to presume lower cognitive ability or other disabilities due to visual impairment.^36^ In interactions with the patient, be considerate of their visual impairment, but do not overly focus on it. Remember, ED visits for blindness and low vision are exceedingly rare^40^; thus, a blind patient is unlikely to be seeking emergency care for their blindness. Treat the blind patient as you would your other patients as much as possible, and do not overly placate the patient. For example, if the blind patient needs to sign a consent form, you can make the necessary accommodations such as reading the form out loud.^29^^,^^32^^,^^33^

### Placement Strategies and Accessibility

#### Optimal Location for Patients in the ED

It is common for people who are VI to have heightened sensory sensitivity, particularly to sound.^41^^,^^42^ This is especially true for people with early vision loss.^43^ Therefore, making considerations for adapting the care environment can contribute to a more comfortable patient experience. For example, placing the patient in the quietest part of the ED can help.^32^^,^^35^ This may also help prevent delirium, particularly if a patient needs to stay in the ED for a prolonged period of time.

#### Ensure Access to Assistive Devices

Accessibility to personal assistive devices, such as mobility equipment, should be considered.^32^ These devices, like canes or guide dogs, are considered an extension of the person and are legally recognized as medical equipment under the ADA. For patients with a guide dog, clinicians and other healthcare staff should understand that the dog has a specific job and, thus, should not be bothered or inhibited. Healthcare staff are not required to directly care for a guide dog but may assist with care tasks if the patient requests and time permits. By ensuring that VI patients have continual access to these aids, we can help facilitate independent navigation and mobility, which serves to preserve their autonomy and reduces potential distress during their stay.^29^^,^^33^^,^^35^

Phones or smartwatches can also help bridge gaps in healthcare equity by serving several functions. For example, VI patients often use speech-to-text software or navigational aids, which they may access through their personal devices.^30^ Many hospitals offer apps or online tools to track appointments, view lab results, or communicate with clinicians. Ensuring access can, therefore, facilitate communication with medical staff and contribute to a more comprehensive understanding of their care. Finally, personal devices enable patients to maintain contact with their social networks, friends, or family, which can help promote emotional well-being during a potentially stressful hospital stay.^30^ Some patients may rely on their devices for entertainment or distraction, which can make the stressful ED environment easier to cope with. In all, maintaining access to personal technology is not merely a convenience for VI patients; it plays a crucial role in ensuring equity and inclusivity by fostering a more patient-centered approach to care and empowering them in the management of their healthcare.^30^^,^^32^ Finally, ensuring that patients are aware of the location and operation of the call light can further empower them and facilitate immediate communication, especially in emergency situations.^30^^,^^32^ These simple strategies may also help prevent delirium in VI patients who are already at higher risk.

#### Guiding Patients

If a VI patient needs to move somewhere (eg, to use the bathroom), and is stable enough to ambulate, it is important to know how to best assist the patient. Allowing ambulatory patients to walk also provides them with autonomy. Guiding can be a daunting task for those who have never done it, but this task is relatively simple. First, the healthcare staff should ask the patient whether they would like a guide and whether they would like to bring their assistive device (ie, cane or guide dog). If they say yes, allow them to stand; then, the healthcare worker should stand next to the patient and tap the patient’s arm. The patient will then take the person’s arm or elbow and will be ready to be guided. The healthcare worker should walk at a normal pace. If the worker is passing through a tight area, they should simply move their elbow behind their back and hold it there. This will signal the patient to walk behind the staff member. When it’s safe for the patient to return to the clinician or healthcare worker’s, the worker should move their elbow back to their side; this will signal it is safe to return to walking by the worker’s side. Although unlikely in the ED, if the healthcare worker encounters a ledge or stairs, they should inform the patient and pause when they get to the area. This will give the patient enough time to gain stable footing. After, walk up or down the stairs at a normal pace. If the ED staff member encounters a door, open the door and ensure the patient has a hand on the door. This will ensure they are able to control when the door closes. If the patient is using the bathroom, assist them in finding the toilet and sink; then leave the bathroom and give the patient privacy. When finished, the patient will let the staff member know, and they can be guided back to their room.^31^^,^^32^

### Ensuring Quality Continuance of Care

#### Optimal Documentation

When treating a blind patient, it is important to note visual impairment as early as possible and as clearly as possible in the chart and/or on the wristband that the person is wearing, for example.^30^^,^^32^ The ideal time to note visual impairment would be during the intake or triage process. The International Classification of Diseases, Rev 9 and 10 codes for Blindness and Low Vision are 369 and H54, respectively. This would enable the downstream healthcare workers to appropriately adjust their care to a patient with visual impairment.

Upon recognizing that the patient is blind, the patient’s chart should be updated to clearly reflect the visual impairment, as per hospital or ED protocol. If your healthcare setting does not have a protocol, you can seek to establish a standardized protocol. Before implementing, consider that the protocol should be implementable across both electronic and paper health records. One example could be an “eye” icon in an electronic health record (EHR) or a colored sticker for paper charts. Additionally, the same-colored sticker can also be applied as a patient wristband. Finally, ensure that the protocol does not overlap or conflict with another existing department/hospital protocol. For example, if your hospital uses a yellow wristband to signify a fall-risk patient, it is best to use an alternate color to signify a patient with visual impairment. Similar signage used for “fall-risk” or infection precautions can be used on the patient’s door, if admitted.^32^

#### Discharge Considerations

During discharge, patients are often given paper copies of their discharge instructions. However, this is not accessible for VI patients. Thus, it is important to find alternative means of providing this information.^29^^,^^32^^,^^33^ Many EHR systems have websites or apps patients can use to access their health information. For example, Epic (Epic Systems Corporation, Verona, WI) uses the MyChart system, which is screen-reader accessible. Screen readers are software natively installed or downloaded onto devices that use the device’s microphone to read out loud what is on screen. The MyChart app can be used with IOS and Android screen readers, Voiceover and Talkback, respectively, and the website can be accessed with JAWS and NVDA, the two most commonly used Windows screen readers. Although it’s impossible to test every EHR, you can reach out to your information technology department to determine whether your system is screen-reader accessible, and if not, to advocate for updates to be made so all patients can access their health records and discharge instructions.

#### Support Medicaid Expansion

States that have expanded Medicaid coverage see a decreased rate of ED visits among disabled patients. This is likely because it decreases the financial burden for disabled patients to seek preventative care.^7^ Importantly, this may also decrease clinician burden. We recommend advocating for Medicaid expansion in your state. This can be done in many ways, such as contacting your member of congress or representatives at your medical society (eg, American Academy of Emergency Medicine). Additionally, hospital financial services or social workers may be able to assist patients in applying to Medicaid.^32^

#### Connect Patients to a Primary Care Physician

It is known that access to a primary care physician (PCP) is associated with significantly reduced ED visits.^44^ For VI patients who have a myriad of unique needs, it is especially important to connect them with a PCP before they are discharged.^32^ This has also been found to decrease recurrent ED visits among disabled patients.^45^

## LIMITATIONS

This review is limited by the lack of data on VI patients in the ED. It is also important to note that disabled individuals’ experiences are varied and highly personal, so the recommendations provided in this paper are general. All data used in this review are retrospective and observational and, thus, subject to the limitations inherent to those study types. More research is needed to determine the shortcomings of ED care of VI patients.

## CONCLUSION

There are a variety of impactful interventions that can improve ED care for visually impaired patients. These interventions are reproducible, not resource-intensive, and profoundly helpful for VI patients in the ED. Like many ED interventions, these recommendations are not static or comprehensive but rather serve the purpose of furthering a much-needed conversation. These recommendations should also be further studied to determine their patient-centered impact, ideally in partnership with national and state organizations representing VI people. Optimal care in the ED for visually impaired patients is optimal care for all patients. Please consider implementing some or all of these interventions and approaching the care of VI ED patients mindfully and intentionally.
